# Endothelial Cell Dynamics in Vascular Development: Insights From Live-Imaging in Zebrafish

**DOI:** 10.3389/fphys.2020.00842

**Published:** 2020-07-22

**Authors:** Kazuhide S. Okuda, Benjamin M. Hogan

**Affiliations:** ^1^Organogenesis and Cancer Program, Peter MacCallum Cancer Centre, Melbourne, VIC, Australia; ^2^Sir Peter MacCallum Department of Oncology, University of Melbourne, Melbourne, VIC, Australia; ^3^Department of Anatomy and Neuroscience, University of Melbourne, Melbourne, VIC, Australia

**Keywords:** vasculogenesis, angiogenesis, lymphangiogenesis, anastomosis, endothelial cell, zebrafish

## Abstract

The formation of the vertebrate vasculature involves the acquisition of endothelial cell identities, sprouting, migration, remodeling and maturation of functional vessel networks. To understand the cellular and molecular processes that drive vascular development, live-imaging of dynamic cellular events in the zebrafish embryo have proven highly informative. This review focusses on recent advances, new tools and new insights from imaging studies in vascular cell biology using zebrafish as a model system.

## Introduction

Vascular development is an early and essential process in the formation of a viable vertebrate embryo. Blood and lymphatic vascular networks are composed of a complex mix of cell types: for example smooth muscle cells, fibroblasts, pericytes and immune cells are intimately associated and even integrated within mature vessel walls (Rouget, 1873; Horstmann, 1952; Nicosia and Madri, 1987; Fantin et al., 2010; Gordon et al., 2010). The inner lining of the vasculature, made up of endothelial cells (ECs), is the earliest part of the vasculature to develop in the embryo and is instructive in recruiting other lineages and cell types as the vascular system matures. Vascular ECs derive from the mesoderm of the gastrula stage embryo and specifically from the lateral plate mesoderm (LPM) (Sabin, 1920; Coffin and Poole, 1988; Zhong et al., 2001). The process by which early undifferentiated mesoderm is progressively restricted in its fate to form ECs has fascinated developmental biologists for decades.

Early studies of developing ECs relied heavily on genetics and lineage tracing approaches in mice and uncovered a wealth of information about how the vasculature forms (reviewed in detail elsewhere; Eilken and Adams, 2010; Welti et al., 2013). However, more recently researchers have begun to delve into the cellular behaviors that drive EC development and to appreciate the importance of cell dynamics in shaping vascular development in the embryo. One especially useful system for studying spatiotemporal events at a cellular level is the zebrafish, which has become a standard model to investigate how the vasculature develops. The use of zebrafish has demonstrated new gene functions and molecular mechanisms that are highly conserved in mammals, including uncovering mechanisms of disease (Hogan and Schulte-Merker, 2017). This field has benefited from significant technological advances that improve the model’s utility for the study of EC biology *in vivo.* New cellular labels have been developed (see Table 1), new imaging modalities applied and the increasing use of biosensors to dissect key cell biological processes has opened up exciting new possibilities. In this review, we investigate recent studies that have used dynamic imaging and *in vivo* cell biology to understand EC development in zebrafish. We highlight key technical achievements and new biology uncovered, we will also give a broad overview of the current toolbox available and we briefly describe potential new future directions.

**TABLE 1:** A toolbox for zebrafish vascular cell biology and selection of recent findings.

Endothelial cell biology applications	Transgenic line name/s	Specific applications for transgenic	Recent biological insights using this strain	References
Cell lineage tracing reporters	*TgBAC(etv2:kaede)^ci6^*	Lineage tracing of angioblasts	Distinct populations of angioblasts give rise to endothelial cells of either the dorsal aorta or the posterior cardinal vein. Angioblasts that originate near the ventral aorta give rise to facial lymphatic endothelial cells. Posterior cardinal vein angioblasts give rise to endothelial cells of the intestinal vessels.	Kohli et al., 2013; Koenig et al., 2016; Eng et al., 2019
	*Tg(msgn1:NLS-Kaede)^pc8^ Tg(msgn1:Cre-ERT2)^pc9^; Tg(actb2:LOXP-AcGFP1-LOXP-mCherry)^pc18^*	Lineage tracing of somitic origins of endothelial cells	A subset of somite cells (endotome) give rise to angioblasts.	Nguyen et al., 2014
	*Tg(EPV.Tp1-Ocu.Hbb2:CreERT2)^jh12^; Tg(fli1:LOXP-Cerulean-Hsa.HIST1H2BJ-LOXP,mCherry)^um43^*	Lineage tracing Notch-active angioblasts	Notch active angioblasts give rise to dorsal aorta endothelial cells but not posterior cardinal vein endothelial cells.	Wang et al., 2011; Quillien et al., 2014
	*Tg(EPV.TP1-Mmu.Hbb:Kaede)^um15^*	Lineage tracing Notch-active endothelial cells	Notch active endothelial cells early in development can give rise to both arterial intersegmental vessel and venous intersegmental vessel endothelial cells. Notch signaling is active specifically in arterial intersegmental vessels even before they anastomose with secondary sprouts to form mature vessels.	Clements et al., 2011; Quillien et al., 2014; Geudens et al., 2019
	*Tg(kdrl:NLS-Eos)^ncv6^ Tg(Kdrl:Dendra2)^cq52^ Tg(fli1a:Gal4FF)^ubs3^; Tg(UAS:Kaede)^rk8^*	Lineage tracing blood vascular endothelial cells	Intersegmental vessel tip cell mitosis results in generation of daughter cells with different size. Later forming angioblasts (25–29 hpf) give rise to endothelial cells in the caudal vessel. Arterial endothelial cells in venous intersegmental vessels migrate dorsally against the flow after secondary sprout anastomosis and are replaced by posterior cardinal vein endothelial cells. Ventral posterior cardinal vein endothelial cells give rise to parachordal lymphatic endothelial cells. Ventral posterior cardinal vein angioblasts give rise to both arterial and venous intestinal vessels.	Hatta et al., 2006; Zygmunt et al., 2011; Fukuhara et al., 2014; Hen et al., 2015; Nicenboim et al., 2015; Costa et al., 2016; Tian et al., 2017; Weijts et al., 2018
	*Tg(lyve1b:Kaede)^nz102^*	Lineage trace ventral aorta angioblasts, and venous/lymphatic endothelial cells	Angioblasts that originate near the ventral aorta give rise to the lymphatic endothelial cells of the facial lymphatics and endothelial cells of the hypobranchial artery.	Eng et al., 2019
Notch signaling reporters	*Tg(tp1-MmHbb:EGFP)^um14^*	Detection of Notch active cells	A subset of angioblasts is Notch active. The tip cell of the venous primordial midbrain channel sprout becomes Notch active prior to fusing with the Notch active arterial system.	Parsons et al., 2009; Quillien et al., 2014; Kaufman et al., 2015; Hasan et al., 2017
	*Tg(EPV.Tp1-Mmu.Hbb.d2GFP)^mw43^ Tg(EPV.TP1-Mmu.Hbb:Venus-Mmu.Odc1)^s^*^940^	Dynamic detection of Notch active cells.	Arterial blood flow promotes Notch signaling in arterial intersegmental vessels. Notch signaling in arterial intersegmental vessel prevents it from secondary sprout anastomoses. The tip cell of venous primordial midbrain channel sprout becomes Notch active prior to fusing with the Notch active arterial system.	Clark et al., 2012; Ninov et al., 2012; Hasan et al., 2017; Weijts et al., 2018
	*TgBAC(dll4:GAL4FF) ^mu106^;Tg(5xUAS:EGFP)^nkuasgfp1a^; Tg(EPV.TP1-Mmu.Hbb:hist2h2l-mCherry)^s939^*	Simultaneous visualization of *dll4* transcription and Notch activation	*dll4* expression is initiated at the tip cell of venous primordial midbrain channel sprout before it becomes Notch active.	Ninov et al., 2012; Hasan et al., 2017
Ca^2+^ signaling reporters	*Tg(10.5xUAS:GCaMP5G)^uq2^ Tg(UAS:GCaMP7a)^zf415^ Tg(UAS:GCaMP7a)^sh392^ Tg(UAS:GCaMP3)^zf350^ Tg(Tol2 fliEbasP::mCherry V2A GCaMP6m)^ric100^*	Visualization of Ca^2+^ signaling	Intersegmental vessel tip cells show Ca^2+^ oscillation that is Vegfa/Kdr/Kdrl-signaling dependent. Intersegmental vessel stalk cells also show Ca^2+^ oscillation. Ca^2+^ oscillation patterns correlate with intersegmental vessel endothelial cell migration and proliferation potential. Calcium signaling increases as dorsal aorta endothelial cells mature. Tmem33 is required for tip endothelial cell Ca^2+^ signaling. The degree of flow-mediated endothelial primary cilia deflection correlates with increased calcium signaling in the dorsal aorta.	Warp et al., 2012; Muto et al., 2013; Goetz et al., 2014; Kita et al., 2015; Yokota et al., 2015; Noren et al., 2016; Lagendijk et al., 2017; Savage et al., 2019
Endothelial cell junction cytoskeleton reporter	*Tg(fli1:LIFEACT-EGFP)^zf495^ Tg(fli1:LIFEACT-EGFP)^mu240^ Tg(fli1:LIFEACT-mClover)^sh467^ Tg(UAS:LIFEACT-GFP)^mu271^ Tg(4xUAS:Has.UTRN-EGFP)^ubs18^*	Visualize F-actin polymerization on F-actin-based structures in endothelial cells such as filopodia and endothelial cell junctions	Filopodia are not required for endothelial cell migration but are essential for tip cell anastomosis. Initial contact site of filopodia (junctional spot) and junctional rings on endothelial cells have high F-actin polymerization. F-actin polymerization is required for retraction of blebs during intersegmental vessel lumenization. Dorsal aorta endothelial cells align in the direction of flow as the dorsal aorta matures. Dynamic F-actin polymerization is observed at the Kuglen “neck.” Arterial endothelial cells in venous intersegmental vessels migrate dorsally against the flow after secondary sprout anastomosis and is replaced by posterior cardinal vein endothelial cells. The common cardinal vein lumenize via lumen ensheathment. The lumen and flow are maintained when endothelial cells in multicellular tube divide. Endothelial cells form multicellular tubes using junction-based lamellipodia.	Helker et al., 2013; Phng et al., 2013; Sauteur et al., 2014; Aydogan et al., 2015; Gebala et al., 2016; Hamm et al., 2016; Sauteur et al., 2017; Sugden et al., 2017; Paatero et al., 2018; Weijts et al., 2018; Kugler et al., 2019; Savage et al., 2019
	*Tg(4XUAS:mClavGR2-Has.UTRN)^ubs27^*	Lineage trace endothelial cell junctions	Endothelial cells form multicellular tubes using junction-based lamellipodia.	Paatero et al., 2018
Endothelial cell junctional protein reporter	*Tg(14XUAS:EGFP-Hsa.TJP1,myl7:EGFP)^ubs5^ Tg(5XUAS:cdh5-EGFP)^ubs12^ Tg(fli1:pecam1-EGFP)^ncv27^*	Visualize ZO1, Ve-cadherin, or Pecam1 localization in endothelial cell junctions.	Junctional spots and junctional rings on endothelial cells have high level of ZO1 and Ve-cadherin. The lumen and flow are maintained when endothelial cells in multicellular tube divide. Endothelial cells form multicellular tubes using junction-based lamellipodia. Intersegmental vessels that remain arterial have a multicellular intersegmental vessel base while intersegmental vessels that will become venous intersegmental vessel have a unicellular base.	Herwig et al., 2011; Lenard et al., 2013; Aydogan et al., 2015; Ando et al., 2016; Paatero et al., 2018; Geudens et al., 2019
Endothelial cell junctional tension sensor	*Tg(cdh5:cdh5-TFP-TENS-Venus)^uq11bh^*	Quantification of Ve-cadherin tension in endothelial cell junctions	Dorsal aorta endothelial cell junctional tension decreases as the dorsal aorta mature.	Lagendijk et al., 2017
Endothelial cell membrane reporter	*Tg(kdrl:HsHRAS-mCherry)^s916^ Tg(fli1:EGFP-CAAX)^md13^ Tg(kdrl:mCherry-CAAX)^y171^*	Visualize EC membrane and filopodia	Lumenization in the intersegmental vessel is flow-dependent. Cerebral vessels form transient Kuglen structure. Filopodia is not required for endothelial cell migration but is essential for tip cell anastomosis. Initial contact site of filopodia (junctional spot) and junctional rings on endothelial cells have high F-actin polymerization. Lumenization in the intersegmental vessel occur through inverse blebbing. Immature cranial vessels are enriched with primary cilia.	Chi et al., 2008; Fujita et al., 2011; Phng et al., 2013; Gebala et al., 2016; Eisa-Beygi et al., 2018; Kugler et al., 2019
Endothelial cell apical membrane reporter	*Tg(fli1:Has.PLCD1-RFP)^md14^*	Visualize endothelial cell apical membrane	Lumenization in the intersegmental vessel occur through inverse blebbing.	Gebala et al., 2016
Endothelial cell golgi reporter	*Tg(fli1:Hsa.B4GALT1-mCherry)^bns9^*	Visualize endothelial cell polarity	Endothelial cells polarize against flow when blood flow is initiated. Arterial endothelial cells in venous intersegmental vessels migrate dorsally against the flow after secondary sprout anastomosis and is replaced by posterior cardinal vein endothelial cells. The difference in endothelial cell polarity between venous intersegmental vessel and arterial intersegmental vessel endothelial cells is pre-determined prior to secondary sprout anastomosis.	Kwon et al., 2016; Weijts et al., 2018; Geudens et al., 2019
Primary cilia reporter	*Tg(actb2:Arl13b-GFP)^hsc5^*	Visualize primary cilia	Immature cranial vessels are enriched with primary cilia. The degree of flow-mediated endothelial primary cilia deflection correlates with increased calcium signaling in the dorsal aorta.	Borovina et al., 2010; Goetz et al., 2014; Eisa-Beygi et al., 2018
Lymphatic endothelial cell fate reporter	*TgBAC(prox1a:KALTA4,4xUAS-E1B:TagRFP)^nim5^*	Visualize *prox1a* expression in endothelial cells	*prox1a*-positive endothelial cells in the posterior cardinal vein undergo mitosis giving rise to a daughter endothelial cell which retains *prox1a* expression and sprout out of the posterior cardinal vein, and a daughter endothelial cell that lose *prox1a* expression and remain in the posterior cardinal vein.	Dunworth et al., 2014; Koltowska et al., 2015; Nicenboim et al., 2015
Hyaluronic acid reporter	*Tg(ubb:SEC-Rno.Ncan-EGFP)^uq25bh^*	Visualize hyaluronic acid localization	Hyaluronic acid turnover in the extracellular matrix is essential for proper Vegfa/Kdr/Kdrl signaling during primary angiogenesis.	De Angelis et al., 2017; Grassini et al., 2018
Cell cycle progression reporter	*Tg(kdrl:mVenus-gmnn)^ncv3^*	Visualize endothelial cells in the S/G2/M phase	Intersegmental vessel endothelial cells leaving the dorsal aorta are in the S/G2/M phase and undergo division shortly after.	Fukuhara et al., 2014
Endothelial cell nuclear reporter	*Tg(fli1:nEGFP)^y7^ Tg(kdrl:nlsmCherry)^is4^ Tg(kdrl:nlsEGFP)^ubs1^*	Visualize EC nucleus	These transgenic lines are widely used to visualize endothelial cell sprouting, migration, division and anastomosis at single cell resolution.	Roman et al., 2002; Blum et al., 2008; Wang et al., 2010

## Cell Behavior and Regulation of Vasculogenesis

Vascular development is typically thought of as occurring in a stepwise manner progressing through vasculogenesis, angiogenesis, lymphangiogenesis, vessel remodeling, and maturation. Vasculogenesis involves the initial formation of the major vessels in the embryonic midline from angioblasts that originate in the LPM (Risau and Flamme, 1995). In zebrafish, this process begins at about 12 h post-fertilization (hpf) and concludes by around 22 hpf with formation of a medial vascular rod (Fouquet et al., 1997; Sumoy et al., 1997). The genetic control of this process has been well defined and is driven by neuronal PAS domain protein 4 like (Npas4l), which is the master regulator of angioblast specification in zebrafish (Reischauer et al., 2016). Npas4l is a transcription factor that regulates expression of early angioblast and endothelial markers *ets1-related protein* (*etv2*), *T-cell acute lymphocytic leukemia 1* (*tal1)* and *fli1 proto-oncogene, ETS transcription factor a (fli1a)*. In addition to genetic analyses, recent studies have used dynamic imaging of cell behavior as angioblasts migrate to the midline to give rise to the dorsal aorta (DA) and the posterior cardinal vein (PCV).

The origin of angioblast cell populations has been a matter of debate: it was unclear if distinct angioblast populations in the LPM are pre-determined as progenitors of the DA and PCV, or if all LPM EC progenitors share the same potential. Using live-imaging of angioblast migration during vasculogenesis with a *Tg(etv2:EGFP)^ci1^* transgenic line, Kohli and colleagues observed that two distinct medial and lateral angioblast pools migrate to the midline separately and sequentially (Kohli et al., 2013). Using *Tg(etv2:kaede)^ci6^*, the angioblast populations could be labeled in a spatially and temporally controlled manner with Kaede (a photoconvertible protein; Ando et al., 2002) and dynamically imaged. This revealed that the medial angioblasts, that migrate to the midline first, predominantly give rise to the DA ECs. The lateral angioblasts, arise later and migrate to the midline to give rise to the PCV ECs (Kohli et al., 2013). A similar observation was made by Helker et al. (2013) when *fli1a*-positive angioblasts were live imaged and lineage traced during vasculogenesis. This demonstrated that the dynamic staging of arterial and venous LPM migration is different and suggests a very early difference between DA- and PCV-generating angioblasts that had not been earlier appreciated. Consistent with these data, lineage tracing of early angioblasts using a *Tg(tp1:creert2)^ih12^;Tg(fli1ep:loxP-nblue-loxP;mcherry)^um43^* transgenic line to label Notch-signaling active ECs, revealed that all Notch active early angioblasts contribute to the DA but not the PCV (Quillien et al., 2014). Similarly, early angioblasts of the arterial system have since been shown to have highly active Erk signaling, suggesting signaling differences in future arterial and venous angioblasts as they depart the LPM (Shin et al., 2016a).

It was long hypothesized that Vascular endothelial growth factor a (Vegfa)/Kdrl (one of two zebrafish VEGFR ohnologs functionally similar to VEGFR2) signaling is essential for angioblast migration (Shalaby et al., 1995; Ferrara et al., 1996). In the zebrafish, notochord-derived Sonic Hedgehog induces *vegfa* expression in the ventral somite, which was proposed to guide angioblast migration toward the midline (Lawson et al., 2002). However, vasculogenesis ensues in both *vegfaa* and *kdrl* mutant zebrafish (Helker et al., 2015; Rossi et al., 2016). In an elegant study that utilized dynamic time-lapse imaging of angioblast migration, Helker and colleagues found that Apelin receptor a (Aplnra), Apelin receptor b (Aplnrb) and a peptide hormone Elabela (Ela) (which binds to Aplnr’s in zebrafish; Chng et al., 2013; Pauli et al., 2014) are required for angioblast migration to the midline (Helker et al., 2015). Angioblasts fail in medial migration in the absence of these key signaling components, while still displaying active filopodial extensions. When *ela* was ectopically overexpressed in notochord mutants lacking *ela* expression, angioblasts preferably migrated toward cells overexpressing *ela*, confirming Elabela as a novel regulator of angioblast medial migration (Helker et al., 2015).

Studies of the dynamic process of angioblast migration from the LPM have significantly improved our understanding of vasculogenesis, yet much remains to be understood. Recently, lineage tracing somite cells using the Kaede protein and Cre/loxP technology revealed that a subset of somite cells termed endotome give rise to angioblasts that colonize the DA (Nguyen et al., 2014). How this movement of cells from the early somite generates angioblasts as a developmental spatio-temporal sequence, requires further investigation. Furthermore, studies have shown that Notch active angioblasts can later give rise to both arterial and venous intersegmental vessels (ISVs), following their incorporation into the DA (Quillien et al., 2014) and movement of ECs can occur between the arterial and venous cords following medial migration in both zebrafish and in mice (Herbert et al., 2009; Lindskog et al., 2014). These studies suggest ongoing refinements following vasculogenesis that remain to be fully understood. Interestingly, late forming angioblasts have been shown to contribute to caudal vasculature between 25 and 29 hpf, after vasculogenesis in the trunk is complete (Fukuhara et al., 2014). In the head, a population of late forming angioblasts have also been found to colonize both lymphatic and arterial vessels (Eng et al., 2019). These studies suggest that apart from LPM and endotome, additional sources of angioblasts may exist to support development of some specific late-forming vessels.

## Cellular and Signaling Mechanisms in Primary Angiogenesis

Once the DA and the PCV are formed at around 22 h post-fertilization, ECs sprout from the DA and migrate to form the ISVs, a process termed primary angiogenesis (Isogai et al., 2003). In this section, recent studies that use live imaging to elucidate EC behavior and signaling dynamics during primary angiogenesis are discussed.

The interplay between Notch and Vegfa/Kdr/Kdrl pathways is essential for proper angiogenic sprouting and the determination of tip/stalk cell identity in sprouting ECs. Vegfa/Kdr/Kdrl signaling induces EC sprouting from the DA, and also stimulates expression of Notch ligand *dll4* in tip cells (Lobov et al., 2007; Jakobsson et al., 2010; Ubezio et al., 2016). This in turn *trans*-activates Notch signaling in trailing stalk cells, which suppresses Vegfa/Kdr/Kdrl signaling (Hellstrom et al., 2007; Leslie et al., 2007; Siekmann and Lawson, 2007; Suchting et al., 2007). This signaling interplay is crucial in angiogenesis but still remains to be understood as a molecular mechanism acting in concert with dynamic cellular behaviors.

**FIGURE 1:**
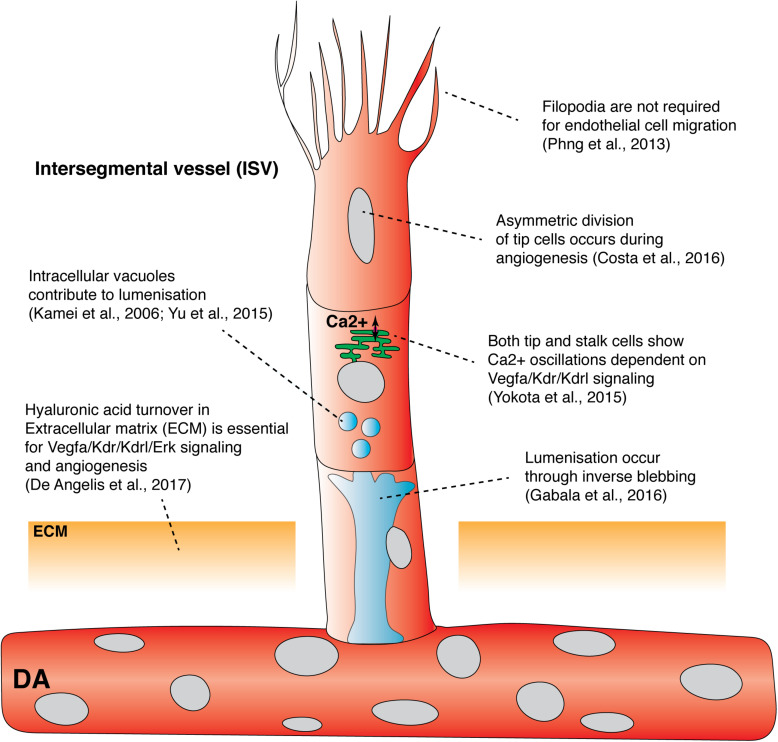
Recent findings from live imaging primary angiogenesis and lumenization in the zebrafish trunk. Recent studies have shown that asymmetric division of intersegmental filopodia have been shown to be dispensable vessel tip cells, hyaluronic acid turnover in extracellular matrix, and Ca^2+^ oscillation in both tip and stalk cells, drive primary angiogenesis. In contrast, filopodia has been shown to be dispensable for endothelial cell migration. Both vacuolar fusion and inverse blebbing have been proposed as mechanisms for lumenization in intersegmental vessels. DA, dorsal aorta; ECM, extracellular matrix; ISV, Intersegmental vessel.

One significant insight into tip cell behaviors came from direct imaging of vascular Ca^2+^ during angiogenesis. Yokota and colleagues generated the *Tg(fli1a:Gal4FF)^ubs4^;Tg(UAS:GCaMP7a)^zf415^* transgenic line, which expresses a Ca^2+^ indicator in ECs (Muto et al., 2013; Yokota et al., 2015). Timelapse imaging revealed that ECs actively budding from the DA display dynamic Ca^2+^ oscillations (Figure 1; Yokota et al., 2015). These oscillations were found to be Vegfa/Kdr/Kdrl signaling dependent, indicating that this model serves as a sensor for Vegfa/Kdr/Kdrl signaling. In this context, it was observed that when neighboring ECs prepare to sprout from the DA, both the sprouting and non-sprouting ECs display Ca^2+^ oscillations. Active Ca^2+^ signaling is only maintained by the EC that sprouts, identifying a previously unappreciated dynamic tip cell selection event. In an additional unexpected turn, high speed imaging revealed that stalk cells also showed Ca^2+^ oscillations as they departed the DA following tip cells. Ca^2+^ signaling increased in intensity as the stalk cells migrated away from the DA (Figure 1). Patterned Ca^2+^ oscillations also occur in cultured mammalian cells and are dependent on VEGFA levels, correlating with distinct EC migration behaviors and proliferation potential *in vitro* (Noren et al., 2016). Savage and colleagues recently showed that transmembrane protein 33 (Tmem33) is required for Ca^2+^ oscillations in sprouting ISV ECs. Tmem33 functions downstream of the Vegfa/Kdr/Kdrl pathway to regulate Notch signaling and Erk phosphorylation (Savage et al., 2019). The precise function of oscillatory Ca^2+^ signaling in angiogenesis remains unclear, but these observations indicate signaling events that correlate with cell behaviors during angiogenic sprouting, while not fitting a simple model of high signaling in tip- and low signaling in stalk-cells. Better live imaging of dynamic signaling events and integration of observations with existing models of tip-stalk cell cross talk is clearly needed.

Live imaging has been used to probe the role of cell cycle progression in primary angiogenesis and revealed links between cell cycle, cell divisions and angiogenic signaling. Using the *Tg(kdrl:mVenus-gmnn)^ncv3^* transgenic line, ECs were fluorescently labeled as they progress through S/G2/M phase of the cell cycle (Fukuhara et al., 2014). This revealed that the majority of ECs that sprout are in the S/G2/M phase as they emerge from the DA and then undergo cell division shortly after sprouting, potentially coupling cell cycle state with cell spouting behaviors. Costa and colleagues live imaged this cell-division event using a transgenic marker that labeled EC nuclei and noticed that tip cells undergo a distinctive cell division event which is followed by a seamless reestablishment of tip/stalk cell hierarchy and motility (Costa et al., 2016). Surprisingly, this was not driven by Notch signaling. Dynamic imaging using a photoconvertible *Tg(kdrl:nlsEos)^ncv6^* transgenic and a GFP-tagged alpha tubulin construct revealed differences in daughter cell size post tip-cell mitosis that were due to asymmetric cytoplasm redistribution (Figure 1). This was caused by the mitotic spindle shifting to the proximal pole of the dividing tip cell. Asymmetry in the size of daughter cells was associated with the tip daughter cell displaying higher Erk-signaling activity than the stalk daughter cell (by staged immunofluorescence staining), potentially indicating asymmetric Vegfr activity between daughter cells. This highly quantitative study suggested that asymmetric cell size and asymmetric Vegfr activity, after tip cell division, re-establishes tip/stalk cell hierarchy to maintain coordinated and uninterrupted migration during primary angiogenesis (Costa et al., 2016).

Switches in EC phenotype and signaling occur rapidly and constantly to accommodate changing cell-cell interactions and extracellular cues as angiogenesis progresses (Jakobsson et al., 2010). Recently, computational modeling of the molecular interactions that drive tip cell selection suggested that a positive feedback mechanism should exist to amplify Vegfa/Kdr/Kdrl signaling and to accelerate EC phenotypic switching (Page et al., 2019). Page and colleagues identified genes that were upregulated by Vegfa/Kdr/Kdrl signaling but downregulated by Notch signaling and identified *transmembrane 4 L six family membrane 18* (*tm4sf18*), which is only expressed in ISV ECs when they are sprouting. To ask whether Tm4sf18 is a novel amplifier of Vegfa/Kdr/Kdrl signaling, embryos were treated with a suboptimal dose of Vegfr inhibitor to force the ECs to rely on the proposed positive feedback mechanism. EC dynamics during ISV sprouting in *tm4sf18* mutants were then analyzed. Consistent with Tm4sf18 being an amplifier of Vegfa/Kdr/Kdrl signaling, *tm4sf18* mutants had delayed emergence of both tip and stalk cells from the DA. *tm4sf18* mutants also showed delayed recovery of Vegfa/Kdr/Kdrl/Erk signaling after brief treatment of Vegfr inhibitor (Page et al., 2019). Tm4sf18 seems likely to work in concert with Notch-Vegfr feedback, and also cellular mechanisms such as the asymmetric division of tip cells, to help orchestrate angiogenesis.

While signaling downstream of Vegfr’s is crucial to control sprouting, recent studies have probed the fundamental mechanics of cellular control of angiogenesis. Tip cells extend many filopodia which were thought to guide vascular migration and patterning (Gerhardt et al., 2003). To visualize filopodia formation in developing ECs, Phng and colleagues generated the *Tg(fli1:LIFEACT-EGFP)^zf495^* transgenic line that expresses F-actin-binding peptide LIFEACT tagged with EGFP in ECs (Riedl et al., 2008; Phng et al., 2013). Dynamic live imaging of ISV development enabled real-time visualization of F-actin polymerization in the filopodia, contractile ring, cell cortex, and cell junctions, revealing distinct F-actin turnover rates in different intracellular compartments (Phng et al., 2013). To determine the role of filopodia in EC migration and guidance, Phng and colleagues inhibited EC filopodia formation using a low dose of Latrunculin B, a toxin that inhibits actin polymerization (Morton et al., 2000). Surprisingly, ECs lacking filopodia were still able to migrate and form stereotypic patterns of ISVs (Figure 1; Phng et al., 2013). Filopodia depleted tip cells were also able to respond to changes in guidance cues in the surrounding tissue. Timelapse imaging revealed that ECs lacking filopodia generated lamellipodia that were sufficient to drive EC migration, albeit at reduced velocity. Although EC filopodia are not required for EC guidance during primary angiogenesis, filopodia are required for tip cell anastomosis as tip cells lacking filopodia failed to form stable connections with neighboring tip cells (discussed in detail below). Taken together, the above imaging studies highlight previously unappreciated mechanisms that control endothelial tip cell behavior, EC-EC communication, signaling and mechanics; essential cell autonomous mechanisms during angiogenesis.

Extracellular factors, extracellular matrix (ECM) and the microenvironment in angiogenesis, have received less attention than EC autonomous mechanisms, with imaging the ECM a particular challenge. Using forward genetic screening, transmembrane protein 2 (Tmem2) was found to be required for both primary angiogenesis and hyaluronic acid (HA) turnover in the ECM (De Angelis et al., 2017). When HA localization was visualized using the HA-binding domain of mouse Neurocan tagged with GFP, *tmem2* mutants displayed ectopic HA accumulation in the ECM around the DA and the PCV consistent with a failure of HA turnover (De Angelis et al., 2017). The use of this HA biosensor for live imaging has also now been applied to live-image ECM in the heart (Grassini et al., 2018). A product of HA depolymerization, the small oligosaccharide fragment o-HA, was shown to be essential for proper Vegfa/Kdr/Kdrl signaling during primary angiogenesis (Figure 1). Importantly, injection of either hyaluronidase or o-HA restored primary angiogenesis in *tmem2* mutants (De Angelis et al., 2017). Consistent with zebrafish epistasis experiments, Tmem2 was recently identified as a long-hypothesized membrane bound Hyaluronidase capable of enzymatic breakdown of long HA but tethered to the cell membrane in mammalian systems (Yamamoto et al., 2017). Thus, *tmem2* mutants demonstrate the importance of correct patterning and function of ECM for angiogenic signaling and vascular development. Developing improved tools to image and study the role of the ECM in angiogenesis is needed.

## The Dynamics and Stochasticity of Secondary Angiogenesis

**FIGURE 2:**
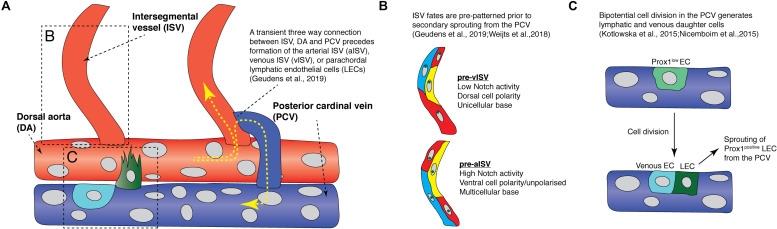
Recent findings from live imaging secondary angiogenesis/lymphatic sprouting in the zebrafish trunk. **(A)** Schematic diagram showing events that occur during secondary angiogenesis and anastomosis in the zebrafish trunk. Yellow arrows indicate direction of blood flow. Red vessels are arteries while blue vessels are veins. Light blue cell is a venous endothelial cell (EC) and the green cell is a fated lymphatic EC. **(B)** Schematic diagram illustrating the difference in Notch activity, cell polarity, and cell arrangements between future arterial intersegmental vessels and venous intersegmental vessels prior to secondary sprout anastomosis. **(C)** Schematic diagram showing how a Prox1-low EC (light green cell) in the posterior cardinal vein undergo bipotential cell division to give rise to a lymphatic EC (green cell) and a venous EC (light blue cell). aISV, arterial intersegmental vessel; DA, dorsal aorta; LEC, lymphatic endothelial cell; ISV, intersegmental vessel; PCV, posterior cardinal vein; vISV, venous intersegmental vessel.

Once ISVs and the DLAV are formed, a second wave of angiogenesis, termed secondary angiogenesis, commences from the PCV from around 32 hpf (Figure 2A; Isogai et al., 2003). Secondary sprouting of ECs from the PCV occurs in a dorsal direction. These sprouts will ultimately give rise to intersegmental veins and the lymphatic vasculature (Isogai et al., 2003; Hogan and Schulte-Merker, 2017). In a remarkably variable process along the trunk of the animal, half of the venous derived sprouts will anastomose with primary ISVs to form venous ISVs (vISVs) that will carry blood. The other half of the secondary sprouts will ultimately not form stable anastomoses but will migrate further dorsally to the horizontal myoseptum and form a parachordal pool of lymphatic endothelial cells (parachordal LECs, PLs), these will go on to form the trunk lymphatic vasculature. The process that decides vISV versus PL identity is not pre-determined by position in the embryo but is stochastic. At any given segmental location in the body plan (except the earliest body segments) a secondary sprout has a 50:50 chance of becoming vISV or PL. The identity of the adjacent vessel as development proceeds is deterministic – the system appears to be patterned as a whole. If a sprout at any position forms a vISV, the probability that the adjacent sprout will now form a PL is vastly increased. ISVs that do not form connections with the PCV remain arterial and are termed arterial ISVs (aISVs) (Isogai et al., 2003; Bussmann et al., 2010).

**FIGURE 3:**
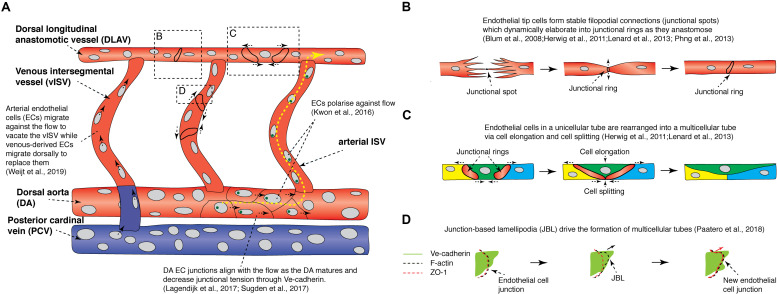
Recent findings from live imaging vessel remodeling and tip cell anastomosis in the zebrafish trunk. **(A)** Schematic diagram showing events that occur during vessel remodeling and tip cell anastomosis in the zebrafish trunk. Yellow arrows indicate direction of blood flow. Red vessels are arteries while blue vessels are veins. Green circles are golgi bodies. **(B)** Schematic diagram illustrating the different steps of tip cell anastomosis. A stable filopodial connection is first established (junctional spot), which elaborates into a junctional ring as the cell-cell junction expands. **(C)** Schematic diagram illustrating how cell elongation and cell splitting occur in the dorsal longitudinal anastomotic vessel to remodel a unicellular tube into a multicellular tube. **(D)** Endothelial cells migrate on top of each other via junctional-based lamellipodia (JBLs) which emanate from the front end of junctional rings. VE-cadherin provides support for F-actin based JBLs to protrude, driving the ZO-1-positive cell junction to migrate forward toward the front end of the JBL via a ratchet-like mechanism. DA, dorsal aorta; DLAV, dorsal longitudinal anastomotic vessel; EC, endothelial cell; JBL, junction-based lamellipodia; PCV, posterior cardinal vein; vISV, venous intersegmental vessel.

Recently, Geudens et al. (2019) showed that ISV fates are pre-determined before secondary sprouting and anastomosis. Live imaging EC polarity in arterial and venous ISVs using the *Tg(fli1:Has.B4GALT1-mCherry)^bns9^* transgenic, which labels endothelial Golgi, revealed that most ECs polarize against flow, resulting in most aISV ECs having a ventral polarity, while most vISV ECs have a dorsal polarity (Figure 3A; Kwon et al., 2016; Geudens et al., 2019). Surprisingly, this polarity difference between arterial and venous ISVs is already established *before* secondary sprouts begin to anastomose to ISVs (Figure 2B; Geudens et al., 2019). There is a high degree of variation and “noise” in cell behavior during the secondary sprouting process. Some secondary sprouts can anastomose with adjacent ISVs but only transiently, forming a temporary three-way circulation between the ISV, DA, and the PCV before disconnecting to then form PLs (Figure 2A). However, ECs that are future aISVs specifically have a multicellular attachment to the DA, but future vISVs have a unicellular attachment to the DA before secondary sprouts begin to anastomose.

Mechanistically, it was found that high Notch activity was only observed in future aISVs even before secondary sprout anastomosis (Figure 2B; Weijts et al., 2018; Geudens et al., 2019). Furthermore, when all secondary sprouting was blocked genetically, future vISVs disconnected from the DA as they would during a normal anastomosis event with a secondary sprout (Geudens et al., 2019). This showed that the state of the initial arterial derived ISVs is pre-determined and will control the ongoing patterning of the mature segmental vasculature. It is unclear if blood flow plays a role in establishing the initial pattern of Notch signaling in the arterial ISVs, with two key studies presenting conflicting data (Weijts et al., 2018; Geudens et al., 2019). How the pattern of aISVs is first determined in the future arteries currently remains unknown. After their formation, as the venous and arterial ISVs mature, all initially DA-derived ECs migrate dorsally against the blood flow and vacate the newly formed vISVs, while venous-derived ECs from the PCV migrate dorsally to replace the arterial ECs over time (Figure 3A; Weijts et al., 2018). This unique system by which different vascular lineages are determined in the zebrafish trunk is highly dynamic, involving complex cell rearrangements, EC-EC interactions, stochasticity and noise.

During secondary angiogenesis, the cells of the future lymphatic system are also established. Recent studies have suggested that LEC fate is beginning to be refined even before secondary sprouting. This process has been reviewed in detail elsewhere (Ulvmar and Makinen, 2016; Hogan and Schulte-Merker, 2017) but there are dynamic cellular behaviors that will be revisited here briefly. In vertebrates, the key marker of LEC fate is the transcription factor PROX1 (Wigle and Oliver, 1999; Wigle et al., 2002). In zebrafish, Prox1 (mainly the *prox1a* homolog) is expressed preceding secondary sprouting in a subset of venous ECs, largely along the dorsal wall of the PCV (Koltowska et al., 2015; Nicenboim et al., 2015; Shin et al., 2016b; Baek et al., 2019). Dynamic imaging using the *TgBAC(prox1a:KALTA4,4xUAS-E1B:TagRFP)^nim5^* transgenic line revealed that a subset of PCV ECs expressing *prox1a* in the PCV display distinctive behaviors preceding sprouting (Koltowska et al., 2015; Nicenboim et al., 2015). Prox1-positive LECs in the PCV undergo division, giving rise to daughter cells with different fates, a dorsally migrating daughter cell that progressively upregulates *prox1a* and a daughter cell that progressively down-regulates *prox1a* expression and remains in the PCV (Figure 2C; Koltowska et al., 2015). This is likely a mechanism that allows for maintenance of sufficient numbers of ECs in the PCV despite departure of ECs during secondary angiogenesis. Interestingly, Prox1 protein is expressed by as many as 65% of sprouts that depart the vein, but only 50% of sprouts will go on to form LECs, suggesting plasticity whereby Prox1 expression can be lost in some ECs, a process not currently understood (Koltowska et al., 2015). A number of new molecular regulators have recently been identified that at least in part control how cells along the PCV are selected to express Prox1 (Koltowska et al., 2015; Nicenboim et al., 2015; Gauvrit et al., 2018; Baek et al., 2019). Nevertheless, understanding how stochasticity of progenitor selection, cell fate plasticity and LEC sprouting are interrelated now calls for a dynamic real time assessment, by imaging the processes as they occur.

## Vascular Lumen Formation, Vessel Remodeling, and Anastomosis

To form a functional vessel that supports blood flow and normal physiological function, angiogenic sprouts must form correct connections with each other, lumenize, and mature. Multiple mechanisms of vascular lumen formation have been proposed in multiple systems and include cell hollowing, which involves intracellular fusion of vacuoles, or cord hollowing, which is driven by EC rearrangement (reviewed in Charpentier and Conlon, 2014; Betz et al., 2016). The exact cellular mechanism governing vascular lumenization in zebrafish has been the source of much debate. Kamei and colleagues used live imaging to show that intracellular vacuoles sequentially fuse to form a single lumen between multiple cells during primary angiogenesis (Figure 1; Kamei et al., 2006). More recently, live imaging of individual ECs expressing membranous eGFP-farnesyl during primary angiogenesis showed that eGFP-farnesyl-positive intracellular vacuoles could fuse to form larger vacuoles present within the cytoplasm of non-lumenized ECs (Figure 1; Yu et al., 2015). These studies suggested vacuolar fusion as a major mechanism in lumen formation.

Gabala and colleagues performed dynamic imaging of the EC membrane in the *Tg(fli1:EGFP-CAAX)^md13^* strain during ISV lumenization. They found that lumen expansion is driven by flow-induced hemodynamic forces that drive formation of highly dynamic apical membrane protrusions into the cell body, a mechanism they termed “inverse blebbing” (Figure 1; Gebala et al., 2016). While inverse blebbing at the lateral sides of an expanding lumen rapidly retracts, blebs that are found at the top of the growing lumen persist, allowing directional expansion of the lumen. Bleb retraction is dependent on actomyosin contraction, with live imaging revealing recruitment of F-actin and Myosin-II to blebs during retraction. Inhibition of actomyosin contraction resulted in excessive and uncoordinated blebbing and resulted in a disorganized, collapsed lumen (Gebala et al., 2016). One further mechanism that can contribute to lumen formation in a different context has been termed lumen ensheathment. The common cardinal vein (CCV) develops over the embryonic yolk in a context where blood flow initially occurs in the absence of an EC-lined blood vessel. Dynamic live imaging of EC migration during CCV development revealed that ECs collectively migrate as a sheet over the initially avascular space created by circulating blood. The ECs eventually enclose this luminal space via lumen ensheathment (Helker et al., 2013). Altogether, it appears that several different cellular mechanisms can be used to facilitate lumenization and that these approaches may differ between vessels of various vascular beds.

In the zebrafish trunk, once ISVs are formed and vascular lumenization commences, tip cells anastomose to form the dorsal longitudinal anastomosing vessel (DLAV) (Isogai et al., 2003). The first step of tip cell anastomosis involves the formation of a stable filopodial connection between the two tip cells (Figure 3B; Herwig et al., 2011; Phng et al., 2013; Sauteur et al., 2017). Timelapse imaging of the *Tg(fli1a:GAL4FF)^ubs3^;Tg(14XUAS:EGFP-Has.TJP1,myl7:EGFP)^ubs5^* line, which expresses the EGFP tagged junctional protein Zona Occludens 1 (ZO1), revealed that the initial contact site between filopodia form ZO1-positive junctional spots. These initial contacts dynamically elaborate into junctional rings as the mutual surface area between the tip cells increases (Figure 3B; Blum et al., 2008; Herwig et al., 2011). A similar cellular mechanism was observed when vascular anastomosis was live imaged during formation of the cranial vascular palatocerebral artery (Lenard et al., 2013). Apart from ZO1, live imaging revealed that these anastomotic junctional spots and rings have high Ve-cadherin and F-actin levels (Lenard et al., 2013; Phng et al., 2013; Sauteur et al., 2017). The formation of a stable connection between tip cells requires VE-cadherin, as *ve-cadherin* mutants fail to form a mature junctional ring but do initiate filopodial connections (Lenard et al., 2013; Sauteur et al., 2017). This results in the formation of multiple ZO1-positive junctional spots in *ve-cadherin* mutants. The adhesion protein Esama is also required for the formation of filopodial connections with *ve-cadherin* and *esama* double mutants failing to form any junctional spots (Sauteur et al., 2017).

Live imaging has thus revealed how junctional rings are formed and how key molecules regulate this process. Following the formation of junctional rings, junctional remodeling and lumenization commence, which involves either ECs migrating over each other to form elongated junctional rings and a multicellular lumen, or lumen extension through via membrane invagination to form a unicellular lumen (Figure 3C; Herwig et al., 2011; Lenard et al., 2013). For a detailed description of this morphogenesis process see Betz et al. (2016). Timelapse imaging of junctional rings during palatocerebral artery (PLA) formation revealed that an initially unicellular lumen is actively remodeled into a multicellular tube by cell splitting, a unique cellular mechanism which encompasses splitting of the EC cytoplasm to allow the adjacent junctional rings to elongate and connect forming a new cell-cell junction between three ECs (Figure 3C; Lenard et al., 2013). Cell splitting was also observed when larger, later forming vessels anastomose, and when unicellular tip cells anastomose with multicellular lumenized vessels. Multicellular tubes provide additional advantages to the organism as blood flow is readily maintained during cell division along the tube (Aydogan et al., 2015). In contrast, the lumen is collapsed and flow is temporarily stopped during EC division in unicellular tubes.

As discussed above, a key step in formation of a mature multicellular tube is elongation of initial junctional rings between ECs (Blum et al., 2008; Herwig et al., 2011; Lenard et al., 2013). High-resolution and high-speed imaging of junctional ring dynamics has shown that junctional rings are associated with oscillating lamellipodia-like protrusions, termed junction-based lamellipodia (JBL) (Figure 3D; Paatero et al., 2018). JBLs emanate from the front end of elongating junctional rings and orient along the vessel axis. F-actin polymerization is observed on protruding JBLs, while Ve-cadherin localization is diffused, covering both the F-actin-based JBL and the surrounding cell membrane (Figure 3D). The ZO1-positive junction is initially localized at the proximal end of the JBL, but at later stages, ZO1-positive junctions gradually move forward toward the front end of the JBL protrusion, forming a new junction (Figure 3D). Based on these observations, Paatero and colleagues proposed that during vessel remodeling and junctional maturation, F-actin-based JBLs form new protrusions that provide adhesive support via intra-endothelial cell adhesions, this drives cell migration along neighboring cells by a ratchet-like mechanism. JBLs also drive DA EC remodeling (described in detail below). F-actin polymerization and Ve-cadherin localization is essential for proper JBL formation and normal JBL oscillation dynamics, ultimately controlling the EC rearrangements that dynamically drive vessel maturation (Sauteur et al., 2014; Paatero et al., 2018).

As embryonic vasculature expands, blood flow patterns are modulated as new vessel connections are made and alternative routes for flow arise (Sugden et al., 2017). In the trunk, maturation and establishment of normal flow patterns in the ISVs from 2 to 3 days post fertilization (dpf) results in lowering of flow velocity and shear stress. The change in flow is accompanied by a reduction in DA and PCV diameter (Lagendijk et al., 2017; Sugden et al., 2017). Live imaging of the architecture of ECs between 2 and 3 dpf has revealed how blood flow modulates DA diameter. DA ECs in 2 dpf embryos have a rounded morphology but DA ECs in 3 dpf larvae are elongated and more aligned with the direction of the flow, resulting in a narrower and more elongated DA (Figure 3A; Lagendijk et al., 2017; Sugden et al., 2017). This change in EC morphology was abrogated in the absence of flow (Sugden et al., 2017). Live imaging of *TgBAC(cdh5:cdh5-TFP-TENS-Venous)^uq11bh^* transgenic vessels, which express a FRET-based Ve-cadherin tension sensor at the junctions, revealed that the Ve-cadherin tension in DA EC junctions decreases progressively during this vessel maturation process (Figure 3A; Lagendijk et al., 2017). Calcium signaling modulates DA EC junctional tension and vessel maturation and the Vegfa/Kdr/Kdrl/Erk pathway also modulates Ve-cadherin tension and DA maturation. Strikingly, TGF-beta pathway component Endoglin, which is associated with arteriovenous malformation (Tual-Chalot et al., 2015), was found to be an essential molecular regulator of both these key cellular rearrangements and overall vessel morphogenesis in the DA during this distinctive transition (Sugden et al., 2017). In mice, corneal ECs lacking Endoglin fail to migrate against the direction of flow resulting in arterio-venous malformation, suggesting that the role of Endoglin in vessel morphogenesis is highly conserved between vertebrates (Jin et al., 2017).

While the mechanosensory mechanisms that detect and transduce flow into cellular responses in endothelium remain to be fully understood, recently the primary cilia on ECs have been examined in detail (Hierck et al., 2008; Goetz et al., 2014; Chen et al., 2017; Vion et al., 2018). Live imaging endothelial cilia using transgenic approaches including the *Tg(actb2:Arl13b-GFP)^hsc5^* transgenic line that expresses ciliary axoneme GTPase ADP ribosylation factor-like GTPase 13B (Arl13b) tagged with GFP revealed that endothelial primary cilia bend in response to flow and local mechanical forces (Caspary et al., 2007; Borovina et al., 2010; Goetz et al., 2014; Eisa-Beygi et al., 2018). Interestingly, loss of cilia in zebrafish mutant models has been reported to lead to intracranial hemorrhage phenotypes (Kallakuri et al., 2015; Eisa-Beygi et al., 2018). In mammals, endothelial cilia dysfunction results in abnormal retinal vessels and increased incidence of intracranial aneurysm (Chapman et al., 1992; Pirson et al., 2002; Rozenfeld et al., 2014; Liu et al., 2018; Vion et al., 2018). Thus, there appears to be evolutionarily conserved functions of cilia in the endothelium between vertebrates. The live-imaging tools developed in zebrafish offer unique opportunities to further explore these functions in-depth.

Overall, the normal morphogenesis and maturation of patent vessels can involve different cellular mechanisms of anastomosis, lumenization, patterned regulation of cell-cell adhesions, remodeling of junctions and key mechanical and signaling regulators. This area of vascular cell biology in particular is benefiting from the unique capacity of the zebrafish model for live imaging cell biology as it happens. Of note, an important step in vascular maturation involves acquisition of mural cell coverage. The use of zebrafish is beginning to shed light on molecular and cellular mechanisms controlling mural cell development in zebrafish, that are conserved in mammals (Jain, 2003; Santoro et al., 2009; Ando et al., 2016, 2019; Stratman et al., 2017). EC-mural cell interactions play many important roles in development and disease and have been reviewed in detail elsewhere (Armulik et al., 2011; Sweeney and Foldes, 2018).

## Heterogeneity in Cellular Mechanisms in Zebrafish Vascular Development

While live imaging of trunk vascular formation in zebrafish has pioneered our understanding of EC dynamics during development, studies in other vascular beds, particularly late forming vessels have been limited. Recent work has uncovered unique cellular and molecular mechanisms required for development of intestinal vessels (Hen et al., 2015; Goi and Childs, 2016; Koenig et al., 2016), coronary vessels (Harrison et al., 2015; Ivins et al., 2015; Gancz et al., 2019; Harrison et al., 2019; Vivien et al., 2019), caudal vessels (Fukuhara et al., 2014; Goetz et al., 2014; Karthik et al., 2018) and the CCV (Helker et al., 2013) among others. Here, we highlight a few recent studies revealing heterogeneity in cellular mechanisms.

In vascular beds in the fin, eyes and the brain, the remarkable observation was made that venous ECs can contribute to arterial vessel formation, highlighting surprising heterogeneity in developmental mechanisms (Bussmann et al., 2011; Fujita et al., 2011; Xu et al., 2014; Kametani et al., 2015; Kaufman et al., 2015; Hasan et al., 2017). During fin regeneration in zebrafish, live-imaging of intubated adult animals revealed that venous tip cells can migrate against the direction of the moving vascular front and integrate into the remodeling artery (Xu et al., 2014; Kametani et al., 2015). Chemokine receptor Cxcr4a is essential for this process as *cxcr4a* mutants lacked arteries in the regenerating fin because venous ECs fail to migrate toward the remodeling artery (Xu et al., 2014). Similarly, in eye development when venous ECs emanating from the primordial midbrain channel (PMBC) were dynamically imaged using Notch activity reporters, stable Notch activation was detected in cells of the venous sprout derived from the PMBC (Kaufman et al., 2015; Hasan et al., 2017). This venous, Notch active sprout from the PMBC fuses to the Notch-active arterial system. *dll4* is first expressed in both tip and stalk cells of the venous sprout before Notch activation commences, and Notch signaling in the tip cell is induced in *trans* by *dll4* in neighboring stalk cells in a mechanism leading to expression of chemokine receptor *cxcr4a* (Hasan et al., 2017). Similar to the fin, Cxcr4a is essential for proper venous EC migration and artery formation as venous sprouts from the PMBC do not fuse with the arterial system in *cxcr4a* mutants. In mice, CXCR4 is expressed in tip cells in developing retinal vessels, and these cells give rise to retinal arterial ECs (Xu et al., 2014; Pitulescu et al., 2017). However, it remains to be understood in detail the degree to which venous derived tip cells are conserved between zebrafish and mammals. More studies are clearly required to investigate how Notch/Cxcr4a signaling is regulated in different vascular beds to drive blood vessel morphogenesis of both early and late forming vessels.

The cerebral vessels in zebrafish develop under the control of brain specific molecular pathways (reviewed in Hogan and Schulte-Merker, 2017). Imaging of developing cerebral vessels revealed the formation of striking, large, transient, vesicle-like membranous protrusions, termed kugeln, that never detach from the parental ECs (Kugler et al., 2019). Kugeln do not have an EC nucleus and contain little cytoplasm, but dynamic F-actin polymerization is observed at the kugeln neck. Similar to cellular blebs, inhibition of F-actin polymerization using Latrunculin B increases kugeln number, while Myosin II inhibition by Blebbistatin reduces kugeln number (Charras et al., 2008; Kugler et al., 2019). Kugeln formation is modulated by various signaling pathways such as Vegfa/Kdr/Kdrl, Notch, and Wnt signaling, but their formation is not dependent on blood flow (Kugler et al., 2019). While a similar structure has not been reported in mammals, this may be due to its transient nature, making it difficult to identify unless live-imaged. Future studies are needed to investigate the function of this unusual cellular component of vessels and whether they are involved in maintaining brain vascular homeostasis.

The formation of the craniofacial lymphatic vasculature occurs in a very different manner to lymphatics in other tissues and provides compelling evidence for heterogeneity in developmental and cellular processes (Okuda et al., 2012; Astin et al., 2014; Shin et al., 2016b, 2019; Eng et al., 2019; Vogrin et al., 2019). For example, Eng and colleagues lineage traced cells that contribute to facial lymphatics using the photoconvertible Kaede protein. They revealed that facial LECs located laterally originate from veins such as the posterior head sinus (PHS) and the CCV, while facial LECs located ventrally are derived from a population of *etv2*-positive angioblasts first detected close to the ventral aorta [termed the ventral aorta angioblasts (VA-As)] (Okuda et al., 2012; Eng et al., 2019). Dynamic timelapse imaging of the *Tg(etv2:EGFP)^ci6^* transgenic strain demonstrated that the VA-As migrate as a string of cells and do not connect to a lumenized vessel prior to connecting to the facial lymphatic sprout (Eng et al., 2019). Unlike all other cells that contribute to the early larval lymphatic network, these angioblasts appear to derive from a non-venous source. Further studies are needed to identify the exact cell origin of these non-venous VA-As. Studies in mice have also proposed non-venous origins of cardiac, dermal and mesenteric lymphatic networks (Klotz et al., 2015; Martinez-Corral et al., 2015; Stanczuk et al., 2015; Pichol-Thievend et al., 2018; Maruyama et al., 2019; Stone and Stainier, 2019).

In the later larval brain, live imaging of the meningeal vessels revealed that the cerebral blood vessels are closely associated with mural lymphatic ECs [muLECs, also known as brain lymphatic endothelial cells (BLECs) and fluorescent granular perithelial cells (FGPs)], which express lymphatic markers such as *lyve1b*, *flt4*, *mrc1a*, and *prox1a* (Bower et al., 2017; Van Lessen et al., 2017; Venero Galanternik et al., 2017). These enigmatic cells do not form patent vessels but rather appear to function as local scavenger cells that clear tissue waste. However, muLECs sprout out from the choroidal vascular plexus at approximately 54 hpf in a process clearly akin to lymphangiogenesis and in a Vegfc/Ccbe1/Flt4 signaling-dependent manner (Bower et al., 2017; Van Lessen et al., 2017; Venero Galanternik et al., 2017). Therefore, a classical angiogenic process appears to give rise to a non-vascular lineage. The mechanisms involved in muLEC formation, why muLECs express lymphatic markers, are driven by lymphatic cues, but do not form lymphatic vessels, remain unclear. One recent study has highlighted a role for muLECs in cerebrovascular regeneration upon injury of local blood vessels (Chen et al., 2019), perhaps suggesting roles for this lineage in tissue repair and regeneration.

Overall, the above examples of venous derived tip cells contributing to arteries, novel vascular structures, unique contributions to developing vasculature and vessels giving rise to potentially non-vascular cells, all serve as examples of the plasticity and heterogeneity of the vasculature. Of particular note, these studies all relied upon the utility of the zebrafish model for live-cell imaging and dynamic *in vivo* cell biology.

## Concluding Remarks

In recent years, a combination of developmental genetics and live cell imaging using the zebrafish as a model system, have uncovered highly conserved molecular mechanisms in vascular development and led to a deeper understanding of the EC phenotype. Deciphering molecular mechanisms in blood and lymphatic vessels during development and pathogenesis has increasingly pushed the boundaries of genetic and genomic studies. This is partly motivated by new techniques and opportunities, such as single cell RNA-seq and CRISPR-based gene editing. While new genetic approaches will allow us to resolve the transcriptional changes associated with single cells and to manipulate any gene in the genome, it will become increasingly important to understand how genetic changes influence the cellular phenotype. Understanding phenotype in physiologically intact *in vivo* systems is essential. With the unique attributes of the zebrafish for cellular imaging, continued advancements in microscopy and the expanding numbers of transgenic tools available, we can expect that future efforts will lead to unprecedented resolution of the cellular and molecular mechanisms controlling dynamic EC development and maturation. Live imaging of EC behaviors, analysis of cellular heterogeneity, organ-specific vessel formation and function is expected to continue to uncover novel mechanisms in the future. Moreover, the development of disease models for a range of vascular pathologies coupled with application of the approaches discussed in this review, offer exciting prospects to understand disease biology and generate new therapeutic avenues.

## Author Contributions

KO wrote the manuscript and designed the figures. BH reviewed and edited the text and figures. Both authors contributed to the article and approved the submitted version.

## Conflict of Interest

The authors declare that the research was conducted in the absence of any commercial or financial relationships that could be construed as a potential conflict of interest.
